# CTX-M-15–Producing *Escherichia coli* in Dolphin, Portugal

**DOI:** 10.3201/eid2112.141963

**Published:** 2015-12

**Authors:** Vera Manageiro, Lurdes Clemente, Daniela Jones-Dias, Teresa Albuquerque, Eugénia Ferreira, Manuela Caniça

**Affiliations:** National Institute of Health Dr. Ricardo Jorge, Lisbon, Portugal (V. Manageiro, D. Jones-Dias, E. Ferreira, M. Caniça);; Centre for the Study of Animal Science/Oporto University, Oporto, Portugal (V. Manageiro, D. Jones-Dias);; National Institute for Agricultural and Veterinary Research, Lisbon (L. Clemente, T. Albuquerque)

**Keywords:** *Escherichia coli*, dolphin, CTX-M-15, fimH30, bacteria, Portugal, antimicrobial resistance

**To the Editor:** The global emergence and pandemic spread of sequence type (ST) 131 CTX-M-15–producing *Escherichia coli* among humans and its detection in livestock, companion animals, and wildlife is a major cause for concern (*1*,*2*). Hence, it is imperative to identify and explore its dissemination traits. If CTX-M-15–producing *E. coli* continues to spread among different environments, therapeutic options in veterinary and human medicine will be greatly narrowed (*1*). *E. coli* is one of the gram-negative bacteria most frequently isolated from bottlenose dolphins (*3*). However, few studies about antimicrobial drug–resistant bacteria in dolphins have been published (*4*–*6*). We explored dissemination linkages between CTX-M-15–producing *E. coli* isolated from a marine dolphin (*Tursiops truncatus*) and clinical isolates collected during the same period from humans all over Portugal.

In 2009, *E. coli* strain LV143, isolated from respiratory exudate collected through the spiracle of a female dolphin from a zoo, was sent to the National Institute for Agricultural and Veterinary Research in Lisbon, Portugal, for bacteriological and mycological analysis and antimicrobial drug susceptibility testing. No clinical history for the animal was available. Mycologic examination detected no fungi or yeasts.

Drug susceptibility testing of the dolphin *E. coli* strain (LV143), performed by the agar dilution method and interpreted according to European Committee of Antimicrobial Susceptibility Testing (http://www.eucast.org/), revealed a non–wild-type phenotype to cefotaxime (MIC >8 μg/mL); it also showed a synergy toward clavulanic acid, suggesting production of extended-spectrum β-lactamase (ESBL). LV143 was also non–wild-type to ampicillin (MIC >64 µg/mL), nalidixic acid (MIC >512 µg/mL), ciprofloxacin (MIC >8 µg/mL), gentamicin (MIC >32 μg/mL), and tetracycline (MIC >64 μ /mL). This isolate remained wild-type to chloramphenicol (MIC 4 μg/mL), florfenicol (MIC 8 μg/mL), sulfamethoxazole (MIC 32 μg/mL), trimethoprim (MIC ≤0.25 μg/mL), and streptomycin (MIC 4 μg/mL).

To analyze the zoonotic potential of the dolphin isolate, we selected 61 human clinical *E. coli* isolates, previously recovered from different specimens during 2004–2009 in 7 geographically separated hospitals in Portugal (Figure), from the National Reference Laboratory of Antibiotic Resistances and Healthcare Associated Infections collection. Inclusion criteria for the clinical isolates were 1) non–wild-type susceptibility to cefotaxime, 2) presumptive phenotypic ESBL production, and 3) genetic similarity by pulsed-field gel electrophoresis. Analysis of the genetic relatedness of human and dolphin isolates, determined by pulsed-field gel electrophoresis that used *Xba*I digested DNA (*7*), revealed 1 major cluster, which included 22 (35%) clinical isolates from 3 regions in Portugal and the isolate from the dolphin (Figure).

**Figure:**
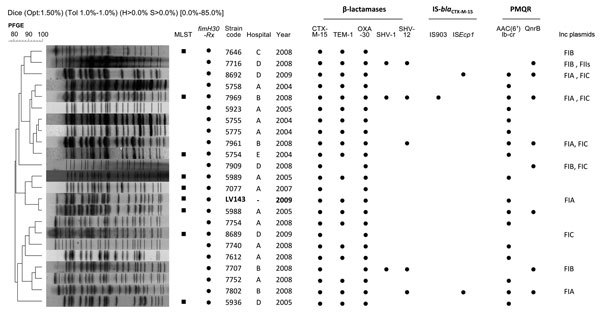
Dendogram of pulsed-field gel electrophoresis (PFGE) profiles showing the relationship between a clonal strain of *Escherichia coli* of animal origin (LV143, in boldface), and 22 *E. coli *isolates from humans. We used the unweighted pair group method and the Dice coefficient with 1.8% optimization (opt) and band position tolerance (tol) of 1%. Isolates with a Dice band–based similarity coefficient of >80% were considered to belong to the same cluster. Black squares under multilocus sequence typing (MLST) indicate sequence type (ST) 131 positivity. Year indicates year of isolation. Black circles indicate fimbral adhesion gene *fim*H, β-lactamase, IS-*bla*_CTX-M-15_, and plasmid-mediated quinolone resistance (PMQR) positivity of indicated combinations. *E. coli* clinical isolates genetically unrelated to the dolphin isolate are not shown. Scale bars indicates percentage relatedness.

The genetic characterization of the 1 dolphin and 22 clinical isolates was performed by PCR and sequencing selective for the most prevalent ESBL-mediated genes (*bla*_TEM_, *bla*_SHV_, *bla*_OXA-G1_, *bla*_CTX-M_) and genes encoding plasmid-mediated quinolone resistance (*qnrA*, *qnrB*, *qnrC*, *qnrD*, *qnrS*, *qepA*, *aac(6’)Ib-cr*), as previously described (*7*). Specifically, the strain recovered from the dolphin contained *bla*_CTX-M-15_, *bla*_TEM-1_, and *bla*_OXA-30_, associated with a plasmid-mediated quinolone resistance gene, *aac(6’)-Ib-cr* (Figure). All clinical isolates were also positive for *bla*_CTX-M-15_ and *bla*_OXA-30_ genes; 18 isolates contained the *bla*_TEM-1_ gene and 3 *bla*_SHV-1_, 5 *bla*_SHV-12,_ 8 *qnrB*, and 16 *aac(6’)-Ib-cr* genes. The presence of class 1 integron, IS*Ecp1*, IS*26*, and IS*903* elements was also investigated, as has been done previously (*8*). The LV143 strain was positive for the insertion sequence IS*Ecp1*, associated with *bla*_CTX-M-15_ (Figure), and was negative for the class 1 integron (data not shown). In 2 clinical isolates, we identified IS*Ecp1*, and in 1 isolate we identified IS*903*. PCR-based replicon typing (*9*) revealed the presence of IncF plasmid group in the 1 animal and 9 human isolates (a selected sample to evaluate PCR-based replicon typing) (Figure).

Multilocus sequence typing (MLST) was performed for 9 of the 23 *E. coli* isolates. According to *E. coli* MLST website (http://mlst.ucc.ie/mlst/dbs/Ecoli), clones from the dolphin and from the humans exhibited the same combination of alleles across the 7 sequenced loci, corresponding to the epidemic ST131, associated with CTX-M-15 and widely disseminated among hospitals in Portugal (*2*,*7*). Within-ST subclones were analyzed on the basis of sequence variation of the *E. coli* fimbrial adhesin gene *fimH*, as previously described (*10*). The *fimH30-Rx* lineage was identified in all 23 *E. coli* isolates (fluoroquinolone-resistant and CTX-M-15–positive isolates), which clustered together on the dendrogram, regardless of MLST result (Figure). It is worth noting that the *bla*_CTX-M-type_ gene has been detected in ESBL-positive *E. coli* isolates from healthy mammals (*1*). 

Our study illustrated clonality among clinical isolates and a dolphin strain with common antimicrobial drug–resistance genes, specifically *bla*_CTX-M-15_ and *aac(6')-Ib-cr*, and common plasmids, such as those from group IncF. These bacteria have gone through identical evolutionary genetic events, which ultimately led to the establishment of the same allelic diversity pattern (ST131 *fimH30-Rx*). The linkage between these 2 reservoirs highlights the zoonotic potential of this isolate from the dolphin.
